# Supplementary figures and images for: Engineered degradation of EYFP-tagged CENH3 via the 26S proteasome pathway in plants
Source: PLoS One. 2021 Feb 12;16(2):e0247015. doi: 10.1371/journal.pone.0247015 (PMC7880479; doi:10.1371/journal.pone.0247015)

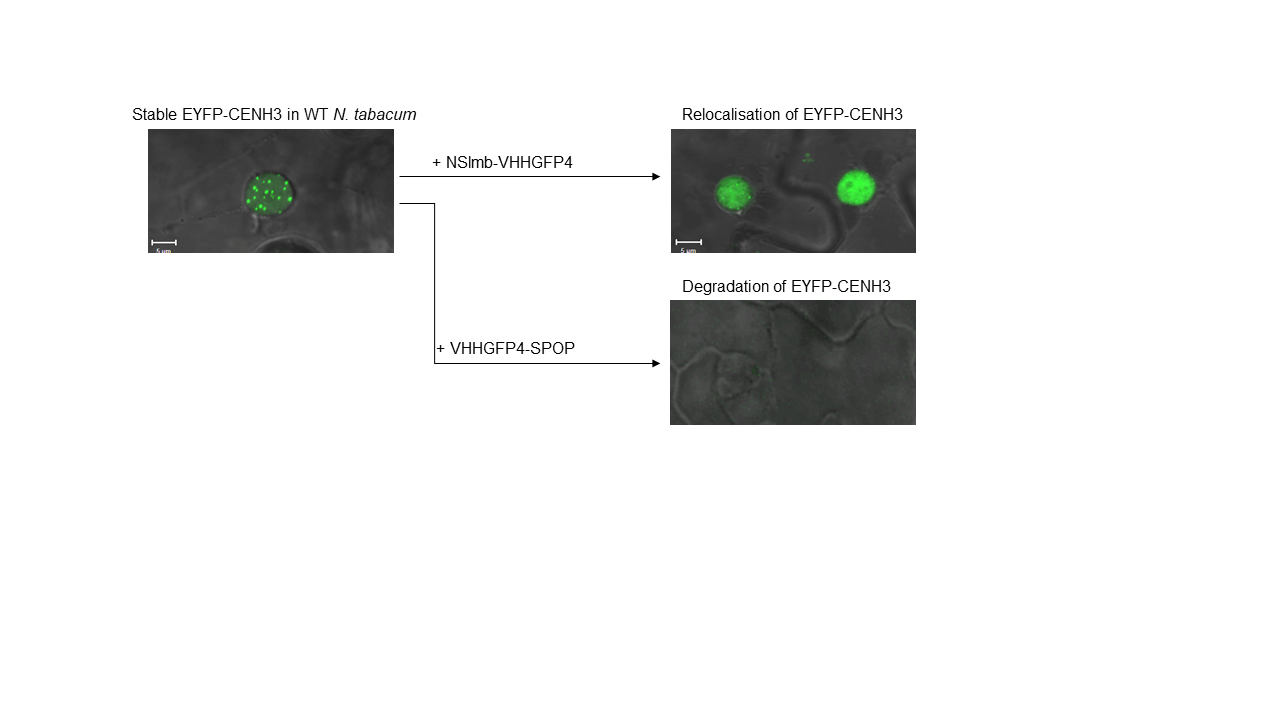

Supplement: S1 Fig — EYFP-CENH3 expressing N. tabacum plants were transiently transformed with either 35S::NSlmb-VHHGFP4 or 35S::VHHGFP4-SPOP. Transient expression of 35S::NSlmb-VHHGFP4 leads to relocalisation of the fluorescence signal into the nucleoplasm. Addition of the VHHGFP-SPOP construct to EYFP-CENH3 expressing plants resulted in complete loss of fluorescence signals. (TIF) [file pone.0247015.s001.tif]

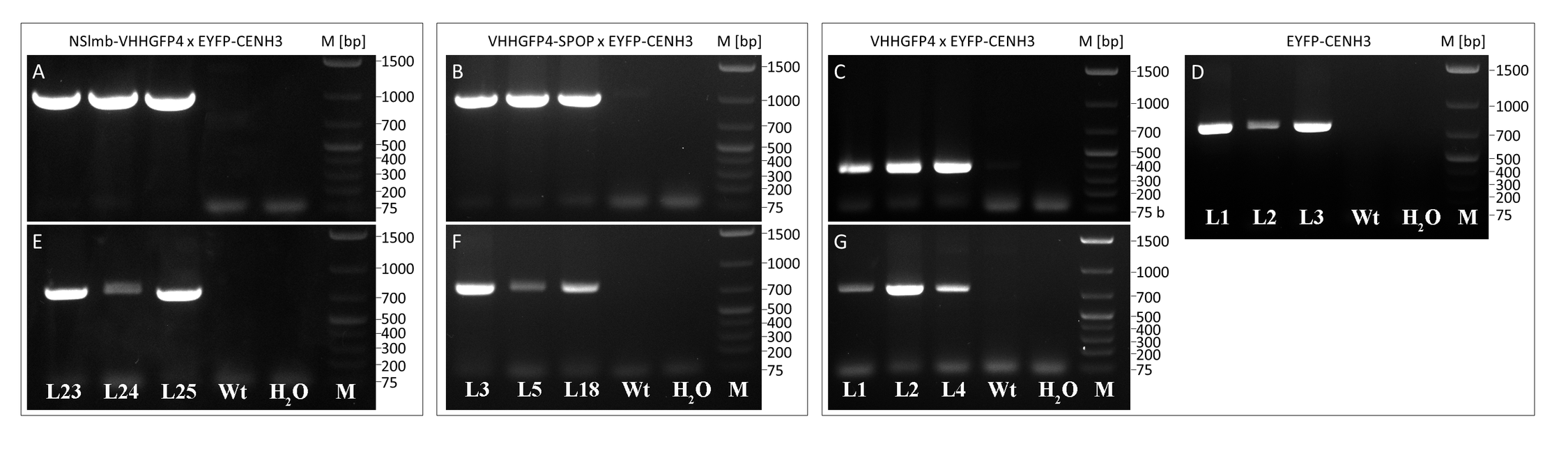

Supplement: S2 Fig — Additional evidence of successful transformation was provided by PCR using genomic DNA and sequence-specific primers (S1 Table). (A) PCR amplified NSlmb sequence in three independently tested transgenic tobacco plant lines with NSlmb-VHHGFP/EYFP-CENH3 overexpression. The calculated length for this construct is 1000 bp. The three lines tested contain the DNA sequence for SPOP expression. (B) Amplified SPOP in transgenic VHHGFP-SPOP/EYFP-CENH3 plant lines. The calculated molecular size of 1000 bp is obtained. The three lines tested contain the DNA sequence for SPOP expression. (C) Amplification of VHHGFP sequences in transgenic VHHGFP/EYFP-CENH3 plant lines. All three tested lines contain the DNA sequence for the nanobody. (D) Amplification of EYFP-CENH3 in control plant lines with EYFP-CENH3 overexpression. The calculated size of 700 bp was confirmed for all three tested plant lines. (E, F, G) Gel separation of the EYFP-CENH3 DNA constructs in NSlmb-VHHGFP4/EYFP-CENH3, VHHGFP4-SPOP/EYFP-CENH3 and VHHGFP4 EYFP-CENH3 transgenic plant lines. 10 individual plant lines were analysed for each transgenic combination. (TIF) [file pone.0247015.s002.tif]

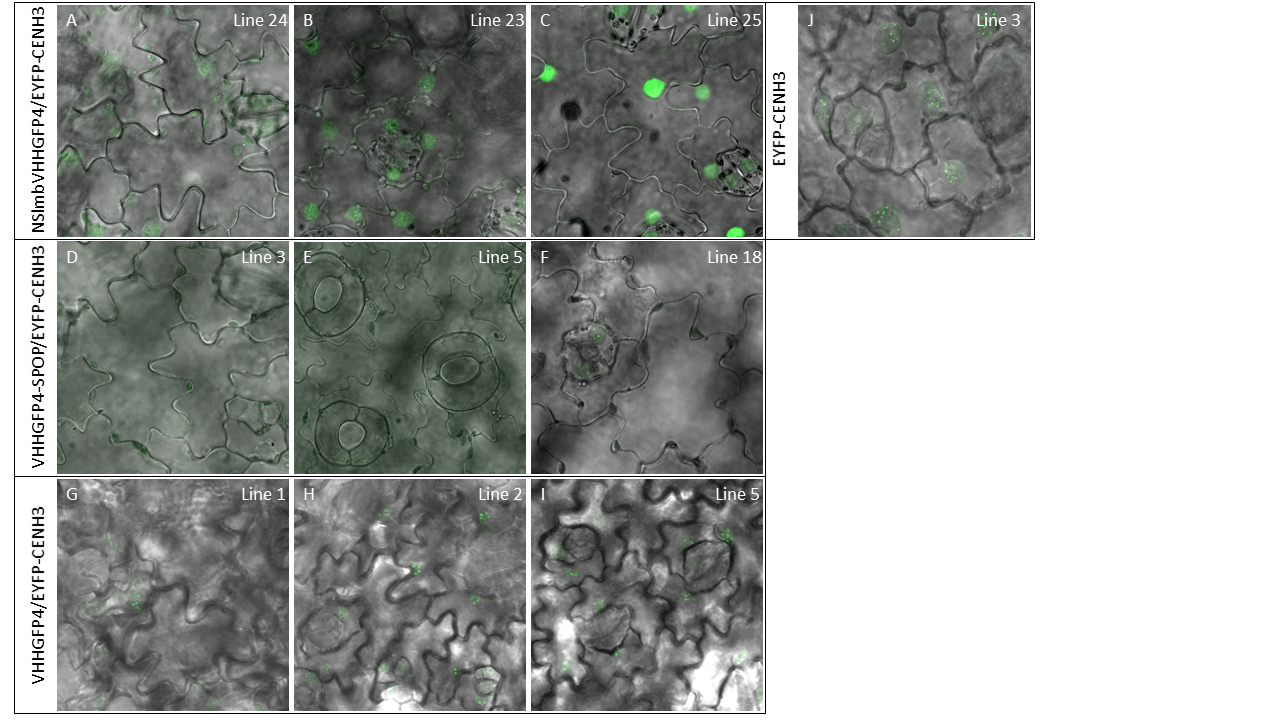

Supplement: S3 Fig — Additional images. (A, B, C) NSlmb-VHHGFP4 EYFP-CENH3 transgenic plant leaf material. Images shown underline the tendency of EYFP-CEN3 to accumulate in the nucleoplasm when co-expressed with the NSlmb-VHHGFP4 construct. Different intensities of the fluorescence signal may result from varying copy numbers of the co-expressed E3-ligase NSlmb-VHHGFP4. (D, E, F) VHHGFP4-SPOP/ EYFP-CENH3 transgenic plant leaf material. Dramatically reduced or a complete lack of specific fluorescence signals was observed in these transgenic plants. Line 18 shows sporadically specific nuclear signals in stomata. (G, H, I) VHHGFP4/EYFP-CENH3 transgenic plant leaf material shows the typical centromeric fluorescence signal of EYFP-CENH3. (J) Control plant with overexpression of only EYFP-CENH3. Images of three independent plant lines are shown. Images were taken at 40x magnification. (TIF) [file pone.0247015.s003.tif]

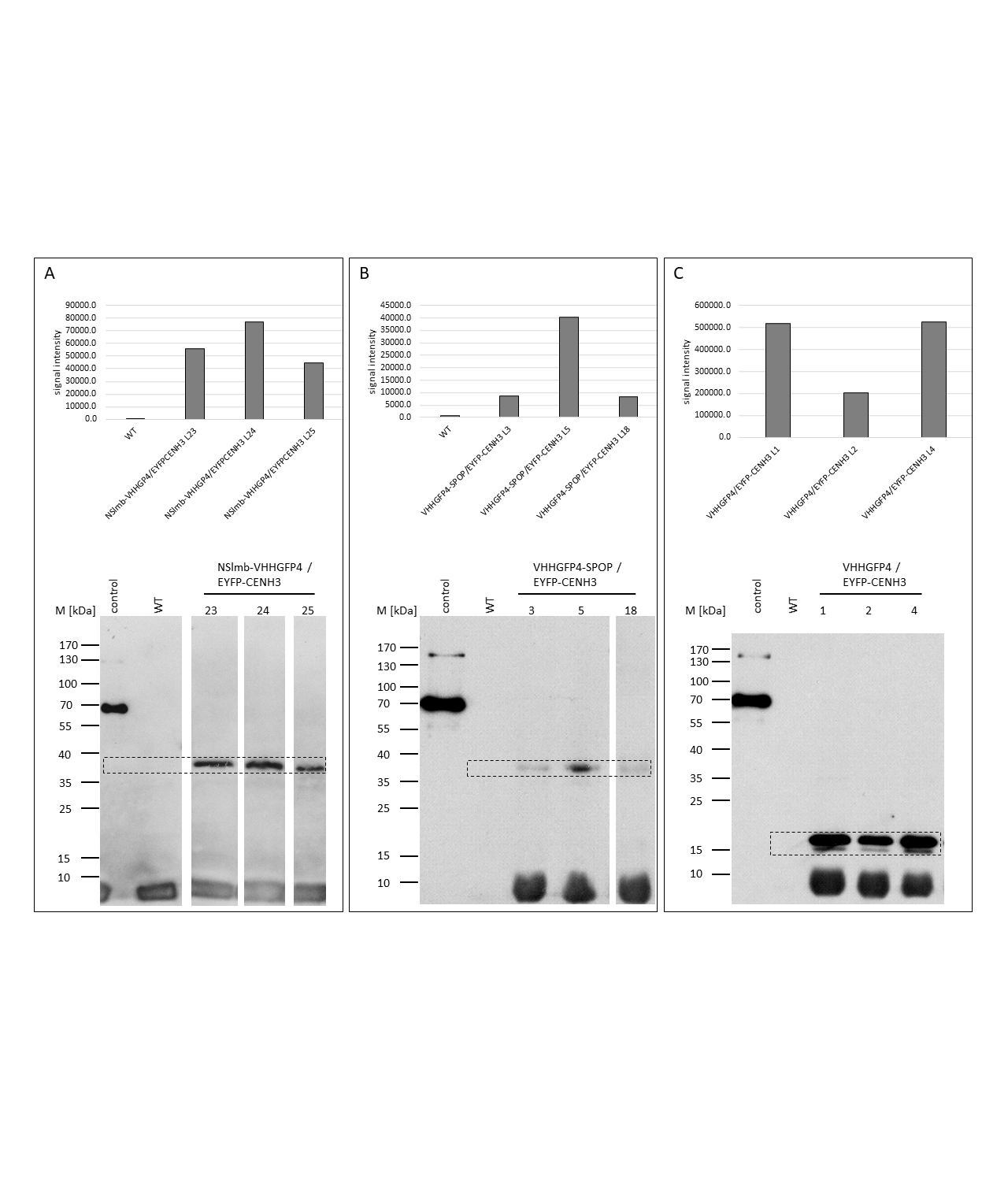

Supplement: S4 Fig — (A) NSlmb-VHHGFP4/EYFP-CENH3 plant lines show a specific signal in the range of 38 kDa, that represents the overexpressed chimeric E3-ligase NSlmb-VHHGFP4. (B) Analysis of transgenic VHHGFP4-SPOP/EYFP-CENH3 overexpressing plant lines. The specific signal at 38 kDa indicates VHHGFP4-SPOP overexpression. (C) Analysed material from transgenic VHHGFP4 / EYFP-CENH3 plant lines. The signal within the range of the 15 kDa marker confirms the overexpression of the anti-GFP nanobody VHHGFP4. Visualisation of the target protein by C-terminal cmyc-tag via a specific monoclonal anti-cmyc antibody (4E10) and ECL-based detection. As internal Western blot control, 100 x ELP protein was used [35]. Faint additional bands below the 10 kDa marker are a result of chlorophyll autofluorescence in the gel running front. Results for three independent transgenic plant lines are shown. Computational quantification of signal strength of single bands was done by “LI-COR Image Studio” software (LI-COR Biosciences–GmbH, www.licor.com) designed for the analysis of Western blot images. (TIF) [file pone.0247015.s004.tif]

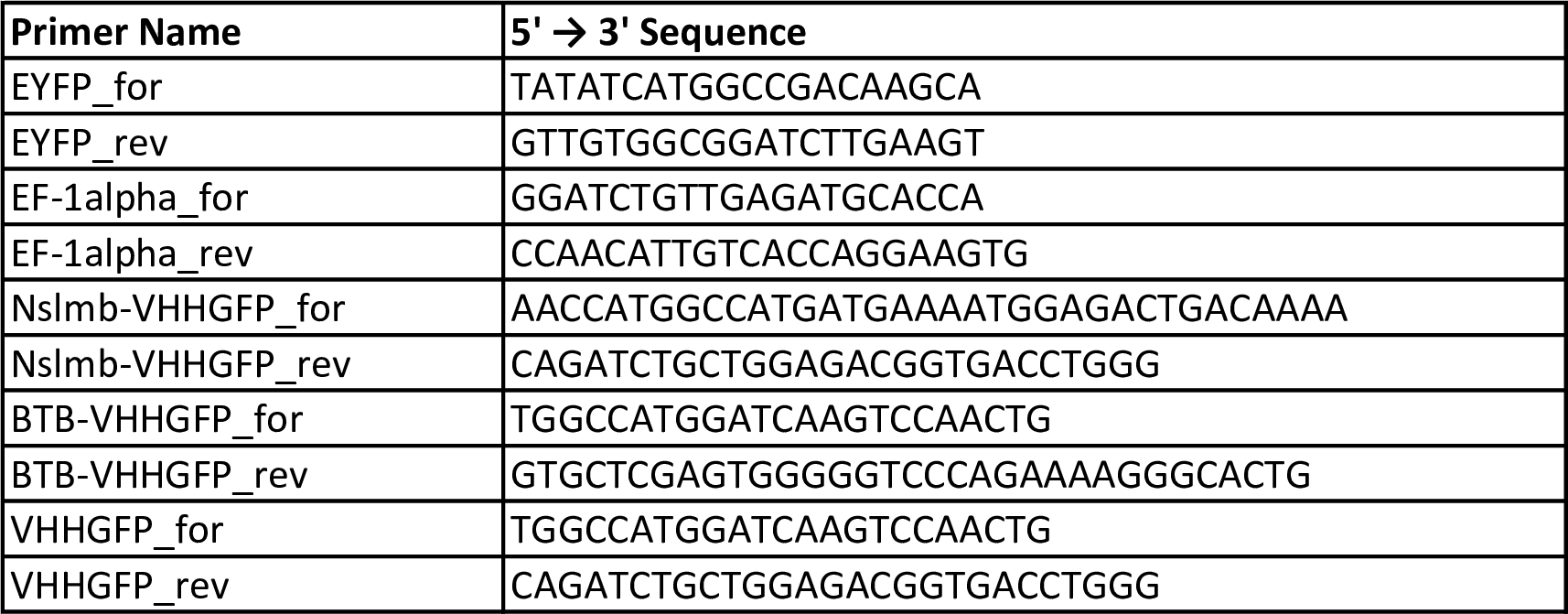

Supplement: S1 Table — The names describe the amplified DNA fragments. Sequences are presented in 5’ to 3’ direction. (TIF) [file pone.0247015.s005.tif]
